# Conical Nanoindentation Allows Azimuthally Independent Hardness Determination in Geological and Biogenic Minerals

**DOI:** 10.3390/ma12101630

**Published:** 2019-05-18

**Authors:** Corinna F. Böhm, Patrick Feldner, Benoit Merle, Stephan E. Wolf

**Affiliations:** 1 Department of Materials Science and Engineering, Institute of Glass and Ceramics, Friedrich-Alexander University Erlangen-Nuremberg, Martensstrasse 5, D-91058 Erlangen, Germany; Corinna.Boehm@gmx.net; 2 Department of Materials Science and Engineering, Institute I, Friedrich-Alexander-University Erlangen-Nuremberg, Martensstrasse 5, 91058 Erlangen, Germany; patrick.feldner@fau.de; 3 Interdisciplinary Center for Nanostructured Films, Friedrich-Alexander University Erlangen-Nürnberg, Cauerstrasse 3, 91058 Erlangen, Germany; 4 Interdisciplinary Center for Functional Particle Systems, Friedrich-Alexander University Erlangen-Nürnberg, Haberstrasse 9a, 91058 Erlangen, Germany

**Keywords:** nanoindentation, biomineralization, hardness anisotropy, mollusks

## Abstract

The remarkable mechanical performance of biominerals often relies on distinct crystallographic textures, which complicate the determination of the nanohardness from indentations with the standard non-rotational-symmetrical Berkovich punch. Due to the anisotropy of the biomineral to be probed, an azimuthal dependence of the hardness arises. This typically increases the standard deviation of the reported hardness values of biominerals and impedes comparison of hardness values across the literature and, as a result, across species. In this paper, we demonstrate that an azimuthally independent nanohardness determination can be achieved by using a conical indenter. It is also found that conical and Berkovich indentations yield slightly different hardness values because they result in different pile-up behaviors and because of technical limitations on the fabrication of perfectly equivalent geometries. For biogenic crystals, this deviation of hardness values between indenters is much lower than the azimuthal variation in non-rotational-symmetrical Berkovich indentations.

## 1. Introduction

Biominerals, i.e., mineralized tissues produced by organisms, are functionally indispensable structures for their host organism as they provide crucial functionality, such as mechanical protection or sensing capabilities. Due to evolutionary-guided optimization over millions of years, they developed excellent mechanical properties exceeding their geological counterparts by combining strength and toughness in the very same material. This originates from a hierarchical and repeatedly graded structural organization overarching multiple length scales. Due to their formidable optimization, biominerals attract considerable scientific attention, and they often serve as a source of inspiration on the quest for new functional material design motifs [1].

Biominerals, such as bones, teeth, shells and other exoskeletal armatures or otoliths, brittlestar eyes, or magnetosomes, are complex and diverse in their chemical composition and crystallographic organization. Beside a varying fraction of organic matrices incorporated into the mineral matrix, whose composition is distinct for a given species, the inorganic part of biominerals can be made from a range of inorganic constituents, e.g., calcium phosphate, iron oxide, silica, and others. Calcium carbonate is probably the most abundant biomineral, and it can be present in a range of polymorphs, i.e., calcite, aragonite, or vaterite. 

**Figure 1:**
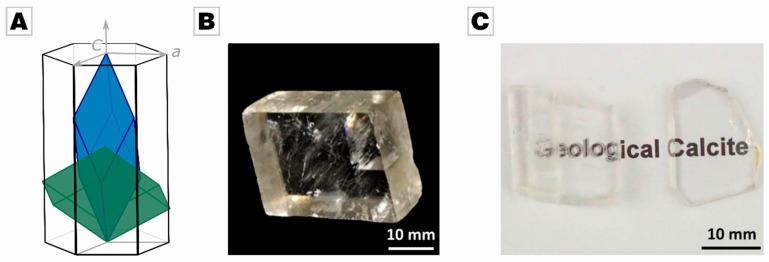
(**A**) Crystallographic unit cells of calcite; elongated rhombohedron (blue) and hexagonal (black) in relation to the cleavage rhombohedron (green). The *c*-axis is the extraordinary axis of calcite. (**B**) A typical calcite rhombohedral as found in nature; the {104} set of planes represent cleavage planes along which calcite readily fractures. (**C**) Birefringence is absent when the optical axis parallel to the extraordinary axis of calcite.

Biomineralizing organisms, such as mollusk and vertebrates, not only exploit chemical and structural gradients in order to enhance the material’s response to external triggers like a mechanical load [1]; they also frequently make use of the anisotropy of the biomineral’s inorganic components. Anisotropy refers to the direction dependence of a material’s properties, e.g., mechanical or optical properties, and originates from the three-dimensional and crystalline organization of the material. By controlling the orientation of anisotropic materials, biomineralizing organisms can optimize, for instance, the mechanical response or functional performance of their biomineralized organs, a capability which is key to withstanding the ever-rising evolutionary pressure they face. A prominent example is calcite, i.e., calcium carbonate in a rhombohedral crystal structure (see Figure 1A). Geological and abiotic calcite is a uniaxial crystal, and its directional birefringence was first reported in 1669 by Rasmus Bartholin [2]. Besides its optical properties, the mechanical properties of calcite also depend strongly on the orientation of the crystal [3]: Hardness, strength, toughness, and Young´s modulus vary with the orientation of a calcite crystal. In 1949, Taylor and Cooke observed different hardness values for different crystal orientations, i.e., the *c*-plane exhibited 105 HV, the cleavage planes {104} showed 136 HV, and the plane perpendicular to the optical axis showed 145 HV [4]. Biomineralizing organisms take this remarkable material anisotropy into account by orienting the most suitable axis towards the direction of attacks of predators. This is why calcite prisms can be found in the exterior layer of many bivalve shells, i.e., *Pinna nobilis*, *Atrina rigida, Ostera puelchana*, *Pteria hierundo* and many others, which point their strong crystallographic *c*-axis towards the exterior [5,6,7,8,9].

**Figure 2:**
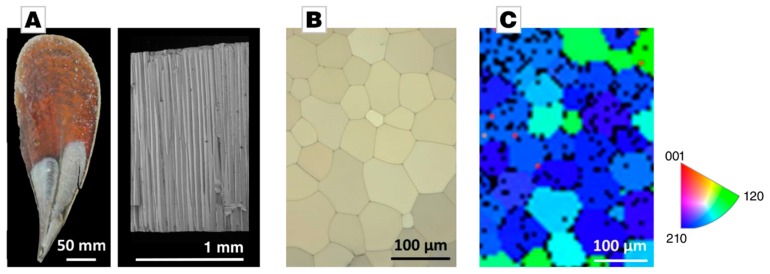
The shell of the Mediterranean Noble Fan shell *Pinna nobilis*. (**A**) On the left, a macrophotograph provides a view on the inner surface of the bivalve shell. On the right, a scanning electron micrograph shows a fracture surface running through the prismatic layer, revealing individual prisms of millimeter length. (**B**) A cross-section of the outer layer shows its prismatic organization with calcite prisms as polygons separated by a thin interprismatic organic membrane; the view axis is parallel to the long axis of the calcite prisms. The micrograph was taken under polarized light before nanoindentation was conducted; the different colors indicate different crystallographic orientations of individual prisms. (**C**) Electron-backscattering diffraction (EBSD, Oxford Instruments, Oxford, UK) mapping was used to determine the azimuthal angle during Berkovich indentation; the inverse pole figure documents the variation of *a_1_*-axis orientation of individual prisms: The prisms colored in blue and green indicate that their a-axis orientation is statistically distributed. The black spots indicate pixels which could not be assigned to a crystallographic orientation because of either insufficient or absent Kikuchi patterns. Some of these black spots represent the amorphous interprismatic organic layer.

The complex graded and hierarchical organization of biominerals, thus requires nanoscale characterization methods. When it comes to probing the mechanical properties of a material on the smallest length scales, nanoindentation is the standard method of choice. When probing an anisotropic material, the geometry of the indenter tip becomes important. An indenter tip that is not rotationally symmetrical with respect to the axis of indentation can cause variations in the determined mechanical properties as a function of the azimuthal angle. This effect is more pronounced in cases of extremely asymmetric indenters, such as Knoop indenters, for which a variation of about 51% is reported [10,11], but also in the case of Berkovich indentation variations of about 7% have been reported [9]. In the case of biominerals, the anisotropic and azimuthally dependent material’s response is even more pronounced due to inhomogeneously incorporated organic matrices [5,6,7,8,9]. The Mediterranean bivalve *Pinna nobilis* might serve as an example (Figure 2A, left). It represents a nacroprismatic bivalve shell, featuring an inner nacreous layer and an outer prismatic calcite layer. The outer layer is composed of tessellating mineral prisms glued together by an interprismatic organic matrix; a fracture surface shows the pronounced aspect ratio of these calcite crystals (Figure 2A, right). A polarized light microscopy image of a polished cross-section, which is oriented perpendicularly to the fracture long-axis of the prisms, shows polygonal calcite crystals separated by a thin and interprismatic organic layer, see Figure 2B. The individual calcite prisms are essentially single-crystalline with their *c*-axis parallel to the long axis of the prisms and orthogonal to the shell’s surface (Figure 2C). The reported hardness values of its prisms range from 3.47 to 4.19 GPa across the literature, although they all have been tested by Berkovich indentation [9,12,13]. Kunitake et al. pointed at this very issue of orientational dependence in Berkovich indentation in a recent study on *Atrina rigida* [9]. They demonstrated that hardness of geological calcite, determined by Berkovich nanoindentation along the *c*-axis, varies by 7% depending on the azimuthal angle. Biogenic calcite prisms of *P. nobilis*, which were indented under otherwise identical conditions, showed an azimuthal hardness variation of up to 20% [9].

The phenomenon of orientationally dependent results during nanoindentation, caused by rotationally asymmetrical indenter tips, leads to incomparable values in the literature since Berkovich indentation is the standard configuration and only rarely is azimuthal dependence considered. This is especially problematic for the field of biomineralization, as hardness is a trait of special importance and it calls for an azimuthally invariant hardness testing method. Spherical indentation could also be an option, but yields depth-dependent properties [14]. In this paper, we provide a straightforward approach to address this issue by using a conical indenter instead. In order to demonstrate the feasibility of our approach, we chose the well-established case of nanoindentation of calcite along its *c*-axis. Beside geological calcite as a standard, we also used biogenic calcite from the prismatic layer of the Mediterranean Noble Fan Mussel *Pinna nobilis*, whose individual prisms are well established to feature near-to single-crystallinity [15,16,17]. We reproduce the finding that conventional Berkovich nanoindentation leads to strong azimuthal dependence in geological and biogenic minerals under otherwise similar indentation conditions. We demonstrate that a conical indenter and a standard Berkovich indenter give comparable results if they have similar projection areas and we further highlight that conical indentation is azimuthally invariant.

## 2. Materials and Methods

### 2.1. Sample Preparation

A geological calcite crystal (Mexico) was mechanically polished, using SiC grinding paper (p320 to p1200) and polishing cloths with diamonds of 6 to 1 µm using a MultiPrep system (Allied, Compton, CA, USA). Final polishing was done using a 40 nm colloidal silica suspension (Allied). In calcite, birefringence is absent if the light path is parallel to the *c*-axis; we used this as a first estimation of the correct orientation and, eventually, confirmed the *c*-axis orientation by electron-backscattering diffraction (EBSD) analysis, which showed an offset of only 1.4°. The *a_1_*-axis was determined by EBSD analysis, and nanoindentation measurements were done rotating the calcite crystal around its *c*-axis, varying the azimuthal angle relative to the *a_1_*-axis. The indents were spaced by 20 times the maximum penetration depth. The outer prismatic layer of *Pinna nobilis* (collected near the coast of Villefranche-s-Mer, Département Alpes-Maritimes, France) was polished parallel to the shells surface, i.e., perpendicular to the prisms’ long axis and thus perpendicular to their *c*-axis. Polishing was performed as described above for the calcite crystal. EBSD analysis confirmed the perpendicular *c*-axis orientation and allowed to determine the orientation of the *a*-axis of each individual prism (see Figure 2C). Nanoindentation was performed in individual prisms of varying azimuthal angles between 0 and 120°.

### 2.2. Nanoindentation Experiments

All nanoindentation experiments were performed in a nanoindenter G200 (KLA, Milpitas, CA, USA), applying the continuous stiffness measurement technique (CSM) [18]. For conventional nanoindentation, a typical Berkovich tip with a projected contact area *A_c_* of ideally 24.5 *h_c_^2^* (with *h_c_* representing the contact depth) and a center-line-to-face angle of 65.3° was used. The hardness was measured as an average over 100 to 250 nm indentation depth. This rather shallow depth was chosen in order to stay below the cracking threshold of the material. At the same time, it was sufficient to produce significantly larger indents than the surface roughness after final polishing. For AFM (atomic force microscopy) imaging, both samples were indented to 250 nm depth; the tip area function was carefully calibrated for the 100 to 250 nm indentation range by performing shallow indentations on a reference fused silica sample, as prescribed by the Oliver-Pharr method. Berkovich indentation was performed at varying azimuthal angles between 0 and 120°, whereas 0° and 120° represents the edge of the indent to be located just right on top of the *a*-axis. At least nine indents were done at a given azimuthal angle in both geological and biogenic calcite. For azimuthal angle independent measurements, a conical indenter was used. For better comparison, a conical indenter with a projected area of contact similar to the one of the Berkovich indenter was chosen. The depth-dependent projected contact area can be expressed as *A_c_* = *πh_c_^2^*tan^2^α, with the included conical angle α [9]. According to this, the semi-angle of a cone indenter needs to be 70.3° in order to be comparable to the Berkovich geometry. In the present study, a cone indenter with a semi-angle of 70.15° and a tip radius of 50 nm was used (Micro Star Technologies, Huntsville, TX, USA), as well as a Berkovich indenter with a specified tip radius of 100 nm (Synton-MDP, Nidau, Switzerland), thus the shallowest indentations are affected by the tip’s rounding [19,20].

## 3. Results

The hardness of the *c*-plane of geological calcite, determined by the average over 100 to 250 nm indentation depth and over all azimuthal angles, shows a value of 2.75 ± 0.05 GPa. The *c*-plane of biogenic calcite, here the calcite prisms of *Pinna nobilis*, is distinctly harder, featuring 4.38 ± 0.32 GPa. The increase by 59% for biogenic calcite, although indented under similar conditions, was caused by a number of strengthening effects, mainly lattice distortions caused by magnesium-ions incorporated into the calcite lattice and intracrystalline organic matrices. Our values coincide well with those reported by Kunitake et al. [9].

**Figure 3:**
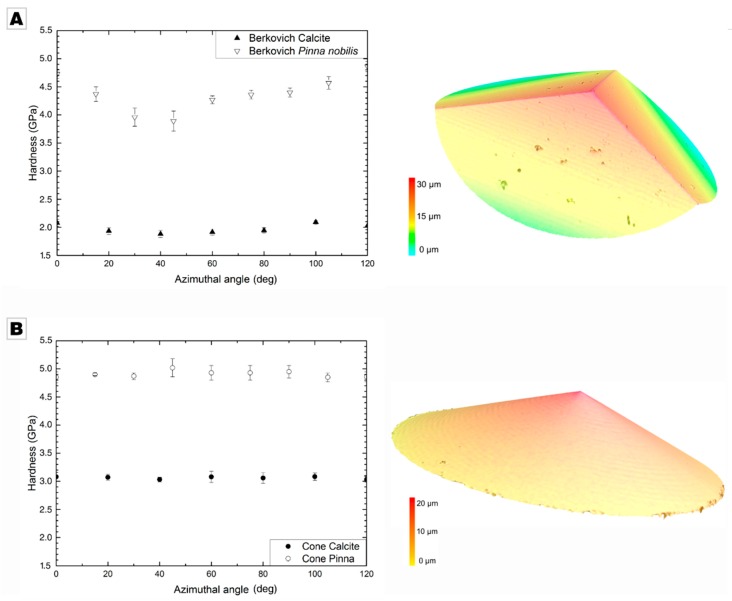
(**A**) Conventional Berkovich nanoindentation of biogenic calcite from *Pinna nobilis* and of geological calcite using a Berkovich indenter (depth: 100 to 250 nm); calcite shows a smaller azimuthal angle dependence compared to the biomineral *Pinna nobilis*. On the right, a 3D laser scanning micrograph of a Berkovich indenter is provided. (**B**) Azimuthal independent nanohardness indentation using a conical indenter in geological calcite and biogenic calcite (indentation depth: 100 to 250 nm). On the right, a 3D laser scanning micrograph of the conical indenter is given.

In both cases, the hardness distinctly varied as a sinusoidal function of the azimuthal angle, i.e., the orientation of the trigonal indenter with respect to the *c*-axis of calcite that features three-fold rotational symmetry. The hardness varied by 4% in geological calcite and 25% in biogenic calcite; the variation was more pronounced in the biogenic mineral due to the inhomogeneously distributed organic occlusions and inhomogeneously incorporated Mg-ions (Figure 3A); a detailed discussion on the origin of this increased azimuthal variance is provided by Kunitake et al. [9]. The azimuthal variation caused the high standard deviation in the averaged hardness value given above. Both minerals showed a minimal hardness at an azimuthal angle of ~40° and the maximal hardness at ~100° (see Table 1). Similar sinusoidal behavior was observed for the modulus, showing an average modulus over all azimuthal angles of 66.05 ± 2.12 GPa in geological calcite and 65.51 ± 1.93 GPa in *Pinna nobilis*.

Using a cone indenter with a similar projection area, the hardness of the *c*-plane of geological calcite, averaged over all azimuthal angles, shows a value of 3.06 ± 0.02 GPa. The *c*-plane of biogenic calcite was, again, distinctly harder, showing a hardness of 4.91 ± 0.05 GPa. The low standard deviation already indicates that, using a radially symmetric indenter, the hardness values were independent of the azimuthal orientation. The standard deviation for geological calcite was reduced by 60% and even by 84% for the calcite prisms of *Pinna nobilis*. The azimuthal invariance is also clearly documented in Figure 3B; the hardness for both geological calcite and biogenic calcite was constant over the whole range of azimuthal orientations; no minima nor maxima were present. We achieved a similar behavior for the modulus with values of 67.35 ± 0.41 GPa for geological calcite and 65.13 ± 0.43 GPa for biogenic calcite, both independent of the applied azimuthal angle.

## 4. Discussion

**Figure 4:**
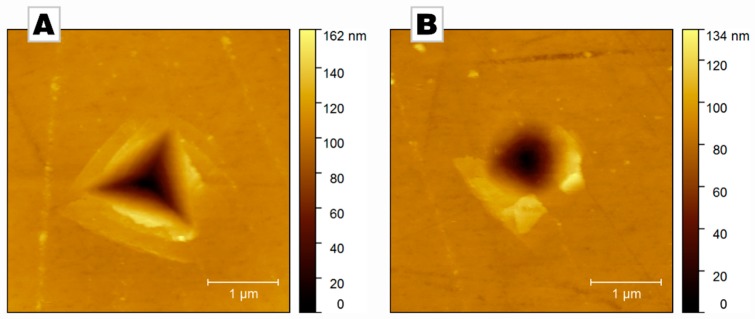
Atomic force micrographs of residual indents in biogenic calcite (*Pinna nobilis*) after indentation up to a depth of 250 nm with (**A**) a Berkovich and (**B**) a conical indenter. Upon unloading, a fraction of this depth is recovered elastically.

Our results clearly demonstrate that conventional indentation experiments using a non-radially symmetric indenter leads to hardness values that are strongly dependent on the orientation of the sample with respect to the indenter. If a radial-symmetric indenter tip is used, e.g., a conical indenter, the orientation dependence is abolished; a result that is both expected and desired. Some significant physical differences can be identified from the topography of the residual indents shown in Figure 4. The asymmetric formation of pile-ups around the conical indenter results from the local crystal orientation of the specimen, which favors specific glide systems [21]: Calcite features some glide systems that have a low critical resolved shear stress and are thus preferentially activated.

This crystallographic dependence is in part lost with Berkovich indentation: Indeed, pile-up formation cannot occur in front of the edges of the pyramidal indenters and is therefore mostly restricted to the center of their faces. Thus, the magnitude of the pile-up formation depends on the in-plane rotation of the punch, which contributes to the strong variations in hardness visible in Figure 3A.

Beside the azimuthally invariant hardness in conical indentation, we additionally observed a distinct increase in hardness of about 11% in geological calcite and 12% in *Pinna nobilis* (Table 2). This might seem unexpected as the opening angle of the conical indenter was chosen to produce projected areas in good accordance with the Berkovich indenter. However, it should be noted that the indentations were performed at a shallow depth (100 to 250 nm), in order to remain below the cracking threshold of the materials [22]. At such depth, the tip blunting of the indenters cannot be neglected as it influences their effective geometries. Therefore, the difference in hardness is possibly a consequence of the different apexes of the punches provided by the manufacturers (specifications: 50 nm for the cone and 100 nm for the Berkovich tip). However, even if the punches had been perfectly sharp, some differences would have been present, as evidenced from Crystal Plasticity Finite Element (CP-FE) simulations used to critically review the equivalence between indentations with a Berkovich and a 70.3° conical indenter [9,23,24,25]. On both metals and ceramics, these studies revealed slight differences in terms of the topology of the plastic zone. Although the load-displacement curves are very similar, a difference in the produced contact stiffness could yield a slight difference in hardness. Finally, we have previously observed that the pile-up formation processes depend on the geometry of the punch, which is also likely to introduce differences in hardness values [26].

All in all, the difference reported in Table 2 between the hardness values from Berkovich indentation and conical indentation is rather slight. More importantly, for comparison purposes, it should be reminded that indentation on a defect-free single crystalline sample—such as the geological calcite specimen—results in a strong indentation size effect (ISE) [27]. In order to compare measurements, it is therefore of paramount importance to select a similar depth range, in order to ensure that differences are caused by intrinsically different properties rather than measurement artifacts.

## 5. Conclusions

In conclusion, we addressed the problem of azimuthally independent hardness determination by nanoindentation by using a conical indenter tip. We hope that our results encourage the use of conical indenters, especially when biogenic or biomimetic minerals are analyzed, in order to enhance and ease the comparison of hardness values across the literature. However, we also demonstrated that the direct comparison of conical and Berkovich indentation should be performed with some caution since slightly different hardness values are to be expected. However, our results show that these differences are small and well below the azimuthal hardness variation. We thus recommend the use of conical indenters, with a Berkovich-equivalent projection area, in order to eliminate the problem of azimuthal orientation dependence when studying and comparing biogenic and biomimetic mineralized matrices. As of yet, only a marginal number of studies rely on conical indentation, e.g., [28,29,30,31,32,33], although Kunitake et al. clearly demonstrated azimuthal variability when indenting a single-crystalline biomineral [9]. Combined with advanced nanomechanical characterization techniques, such as statistical indentation analysis technique introduced by Ulm et al. [34], this will improve our capabilities in chartering the structure-property-relationships in biogenic mineralized matrices, unlocking these resources for bio-inspired materials design.

## Figures and Tables

**Table 1:** Characteristic hardness parameters of geological and biogenic calcite as determined by conventional Berkovich nanoindentation (depth: 100 to 250 nm). Both calcite variants show a relatively high standard deviation in the averaged hardness value, which originates in the azimuthal angle dependent hardness. A minimum hardness was observed at an azimuthal angle of 40° and at maximum hardness at an angle of 100°.

Hardness Values	Average	Minimum	Maximum
Azimuthal angle	0–120°	~40°	~100°
Geo-Calcite	2.75 ± 0.05 GPa	2.72 ± 0.10 GPa	2.83 ± 0.07 GPa
Bio-Calcite	4.38 ± 0.32GPa	3.89 ± 0.18 GPa	4.86 ± 0.09 GPa

**Table 2:** Comparison of hardness for Conical and Berkovich indentation (depth: 100 to 250 nm).

Hardness Comparison	Conical Indentation	Berkovich Indentation
Geological calcite	3.06 ± 0.02 GPa	2.75 ± 0.05 GPa
Biogenic calcite	4.91 ± 0.05 GPa	4.38 ± 0.32 GPa
